# A Case of Extranodal Testicular Involvement in Non-Hodgkin’s Lymphoma Detected through ^18^F-FDG PET-CT

**DOI:** 10.5334/jbr-btr.833

**Published:** 2015-09-15

**Authors:** B. Coulier, L. Montfort, F. Richelle

**Affiliations:** 1 Department of Diagnostic Radiology, Clinique St Luc, Bouge (Namur), Belgium; 2 Department of Internal Medecine, Clinique St Luc, Bouge (Namur), Belgium; 3 Department of Nuclear Medecine, Clinique St Luc, Bouge (Namur), Belgium

A 69 year-old patient was admitted for constipation and with a three days history of left lumbar pain radiating into the left iliac fossa. Contrast enhanced abdominal CT (not illustrated) demonstrated a large retroperitoneal conglomerate (7 × 10 cm) of lymph nodes developing around the abdominal aorta from the level of the renal hilum to the iliac arteries bifurcation. Additional chest CT (not illustrated) confirmed another large conglomerate of enlarged lymph nodes in the superior mediastinum. Smaller nodes were also found in the cervical thoracic angles. The definite diagnosis of diffuse large B cell non Hodgkin lymphoma suggested by CT was confirmed by surgical biopsy of a cervical node. Complementary ^18^F-FDG PET-CT not only confirmed the typical high FDG uptake of the cervical, mediastinal (black arrows on Fig. A) and retroperitoneal (white arrow on Fig. A & Fig. B) of lymph nodes but unexpectedly revealed a high FDG uptake of the right testis (black arrow Fig. B) suggesting associated extranodal testicular extension. Colour Doppler Ultrasound of the moderately enlarged but clinically painless right testis revealed a typical hypoechogenicity of the organ (white star on Fig. C) with massive hypervascularity. Associated swelling and hypervascularity of the epididymis (black star on Fig. C) and vas deferens (grey arrow on Fig. C) were found. The patient is actually treated with classical CHOP chemotherapy regimen and drastic reduction of testicular hypervascularity was already found after 3 courses of chemotherapy (not illustrated)

**Figures A–C:**
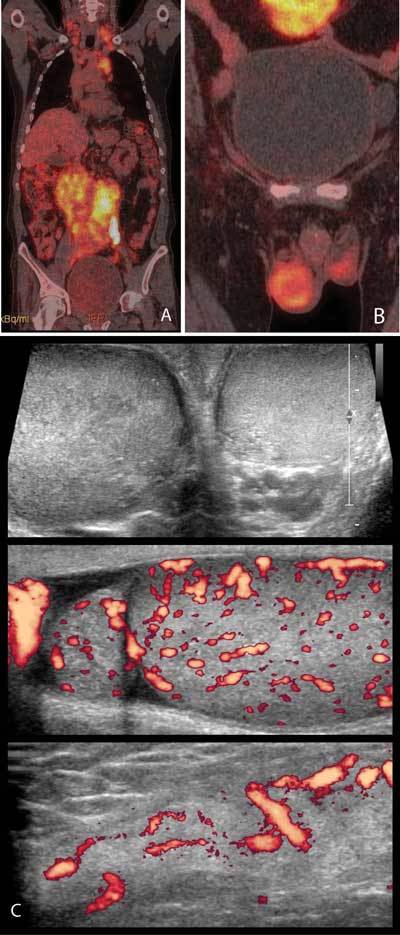


## Comment

Lymphoma arise from the lymphoids cells of the immune system at different stages of differentiation. Thus they may present with a wide range of different clinical, morphological and immunologic features. Currently the term leukemia refers to the primary involvement of bone marrow and blood and the term lymphoma refers to solid tumors of the immune system.

The involvement of extranodal sites (ENS) is a common feature in the course of non Hodgkin’s lymphoma (NHLs). Any organ can be involved with a predilection for the gastrointestinal tract (15–20%) and the bone marrow (15–20%). Other potentially affected organs comprise brain, testis, ovary, lung, nasopharynx, soft tissue, thyroid, kidney, liver, breast, skin, etc. Diffuse large B cell lymphoma (DLBCL) is the most common type of NHL with a prevalence of 33%. The patients may present with primary disease of lymph nodes or that of ENS and more than 50% of patients present with some site of ENS at the time of initial diagnosis.

Testicular lymphoma is a rare entity accounting for less than 1% of all malignant lymphoma and for 1–9% of testicular tumors. However in patient older than 60 years it is the most common testicular tumor (45%) and the most frequent histological type is diffuse large B cell non Hodgkin lymphoma (DLBCL). Testicular lymphoma presents with three distinct expressions: as a rare primary extranodal lesion, as the primary manifestation of occult generalized lymphoma or a as secondary testicular involvement developing during the clinical course of generalized lymphoma (the most common presentation as reported in our patient). Autopsy studies have found microscopic involvement of the testis in 18,5% of NHLs. Thus when a testicular tumor is found in an elderly male or a patient already affected by malignant lymphoma the hypothesis of testicular lymphoma is strongly obvious.

Ultrasound findings include focal or diffusely decreased echogenicity of a generally enlarged and painless testis and diffuse increased vascular flow is found within the mass with Colour Doppler ultrasound. The most important differential diagnosis concerns seminoma and is sometimes difficult even with MRI. Nevertheless lymphoma more commonly also involves the epididymis and the spermatic cord as reported in our patient.

The role of positron ^18^F-Fluorodeoxyglucose positron emission tomography-computed tomography (^18^F-FDG PET-CT) has now been extensively established in the initial staging and in the evaluation of the therapeutic response of Hodgkin and NHLs. The reported case also highlights the role of ^18^F-FDG PET-CT in the detection of unusual or unsuspected extranodal NHLs sites that may have an important bearing on the management and prognosis.

## Competing Interests

The authors declare that they have no competing interests.
